# A new species of *Simulium (Nevermannia)* (Diptera, Simuliidae) from Thailand, with keys to members of the Simulium feuerborni species-group in Thailand

**DOI:** 10.3897/zookeys.89.761

**Published:** 2011-04-11

**Authors:** Takaoka Hiroyuki, Srisuka Wichai

**Affiliations:** 1 *Institute of Biological Sciences, Faculty of Science, University of Malaya, Kuala Lumpur, 50603 Malaysia*; 2 *Department of Infectious Disease Control, Faculty of Medicine, Oita University, Yufu City, Oita, 879-5593 Japan*; 3 *Entomology Section, Queen Sirikit Botanic Garden, P.O. Box 7, Maerim, Chiang Mai, 50180*

**Keywords:** *Simulium*, black fly, Thailand

## Abstract

Simulium (Nevermannia) maeaiense **sp. n.** is described on the basis of female, male, pupal and larval specimens collected from Chiang Mai Province, Thailand. This species is assigned to the *feuerborni* species-group of the subgenus Simulium (Nevermannia), and is distinctive among this species-group in having the female cibarium furnished with numerous dark minute conical processes on the lower part, the female genital fork with a strongly sclerotized horizontal bar on each arm, and six long pupal gill filaments arising nearly at the same level from the common basal stalk and lying in a horizontal plane. Identification keys to seven species of the *feuerborni* species-group reported from Thailand are provided for females, males, pupae and mature larvae.

## Introduction

The Simulium feuerborni species-group within the subgenus Nevermannia Enderlein, redefined by Takaoka (2003), is a small homogeneous group consisting of 24 named and 1 unnamed species (Adler and Crosskey 2010). The majority of species of this group are distributed in the Oriental Region and only 4 are known in eastern parts (China, Korea and Japan) of the Palaearctic Region. This group is relatively rich in species diversity in Thailand, being represented by 5 endemic species: Simulium (Nevermannia) fangense Takaoka and Choochote, Simulium (Nevermannia) fruticosum Takaoka and Choochote, Simulium (Nevermannia) chiangklangense Takaoka and Choochote, Simulium (Nevermannia) vessabutrae Takaoka and Srisuka, and Simulium (Nevermannia) wichaii Takaoka (Takaoka and Choochote 2005,^ 2006^; Takaoka and Srisuka 2010a,b) and one common species: Simulium (Nevermannia) feuerborni Edwards, which was originally described from Java (Edwards 1934) and later recorded from Peninsular Malaysia (Takaoka and Davies 1995), Thailand (Kuvangkadilok et al. 1999) and Sumatra (Takaoka et al. 2000).

In Thailand, we collected one more species of the *feuerborni* species-group, which is easily distinguished from all the known species by the female cibarium, the female genital fork, and the arrangement of the pupal gill filaments. It is described here as a new species based on female, male, pupal and mature larval specimens.

Identification keys to seven species of the *feuerborni* species-group reported from Thailand are also provided for females, males, pupae and mature larvae.

The terms for morphological features used here follow those of Takaoka (2003). Holotype and paratype specimens of the new species are deposited at the Entomology Section, Queen Sirikit Botanic Garden, Chiang Mai, Thailand.

## Systematics

### Simulium (Nevermannia) maeaiense sp. n.

urn:lsid:zoobank.org:act:C50EEB07-3FC6-479F-B1AB-73F914D0DF26

#### Description.

##### Female.

**Figure 1.**
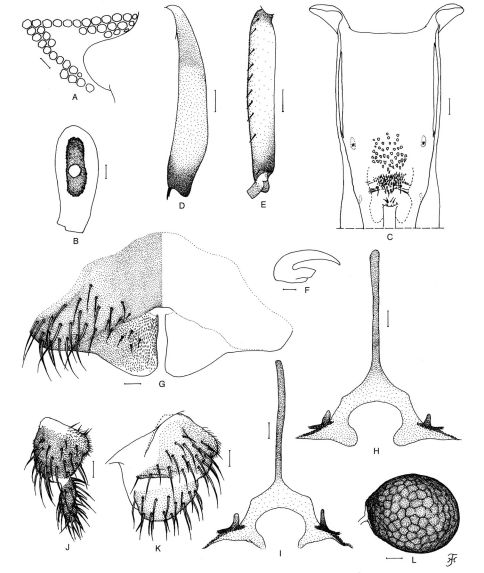
Female of Simulium (Nevermannia) *maeaiense* sp. n. **A** fronto-ocular area (right side) **B** 3rd segment of left maxillary palp with sensory vesicle (front view) **C** cibarium **D** left hind tibia (outer view) **E** basitarsus and 2nd tarsomere of left hind leg (outer view) **F** claw **G** 8th sternite and ovipositor valves (ventral view) **H, I** genital forks (ventral view) **J, K** right paraprocts and cerci (**J** ventral view; **K** lateral view) **L** spermatheca (lateral view). Scale bars 0.1 mm for D and E; 0.02 mm for A–C & G–L; 0.01 mm for F.

Body length 2.4–2.6 mm. Head. Slightly narrower than thorax. Frons dark brown, thinly whitish-gray pruinose, densely covered with whitish-yellow recumbent hairs interspersed with several dark brown longer and stouter hairs along each lateral margin. Frontal ratio 1.55–1.79:1.00:2.53–2.76. Frons-head ratio 1.00:5.00–5.54. Fronto-ocular area (Fig. 1A) well developed, triangular, directed laterally and slightly upward. Clypeus dark brown, whitish-gray pruinose, densely covered with whitish-yellow recumbent hairs (except portion near upper margin bare) intermixed with several dark longer and stouter hairs on each side. Labrum 0.88–1.05 times as long as clypeus. Antenna composed of scape, pedicel and 9 flagellomeres, dark brown except scape, pedicel, and base of 1st flagellomere yellow. Maxillary palp consisting of 5 segments, proportional lengths of 3rd, 4th, and 5th segments 1.00:0.81–0.89:1.46–1.56; 3rd segment (Fig. 1B) much enlarged; sensory vesicle (Fig. 1B) elongate, 0.51–0.53 times as long as 3rd segment, with medium-sized opening. Maxillary lacinia with 7 or 8 inner and 10–13 outer teeth. Mandible with 21–23 inner teeth and lacking outer teeth. Cibarium (Fig. 1C) with 40–44 dark minute conical processes with pointed apices as well as numerous minute spinous processes near lower margin. Thorax. Scutum light to medium brown except anterolateral calli ocherous and narrow portion along each lateral margin and part of prescutellar area dark brown, with 3 dark brown narrow longitudinal vittae (1 medial, 2 submedial), thinly whitish-gray pruinose with 3 dark narrow non-pruinose longitudinal vittae (1 medial, 2 submedial) when illuminated at certain angle of light, and densely covered with whitish-yellow recumbent hairs intermixed with several dark brown upright hairs on prescutellar area. Scutellum ocherous, with several dark brown upright hairs as well as whitish-yellow shorter hairs. Postnotum medium to dark brown, whitish-gray pruinose and shiny when illuminated at certain angle of light, and bare. Pleural membrane bare. Katepisternum longer than deep, dark brown, and bare. Legs. Foreleg: coxa yellow; trochanter dark yellow; femur dark yellow to light brown with apical cap dark brown; tibia dark brown except median large portion of outer surface light brown; tarsus dark brown; basitarsus slender, slightly dilated, 8.4 times as long as its greatest width. Midleg: coxa medium brown; trochanter yellow; femur yellow with apical cap dark brown; tibia light brown except subbasal portion and apical cap medium to dark brown; tarsus dark brown. Hind leg: coxa light brown; trochanter yellow; femur yellow with apical cap dark brown; tibia (Fig. 1D) light brown except extreme base yellow, subbasal portion medium brown and apical cap dark brown; basitarsus (Fig. 1E) grayish-yellow except base and apical portion medium to dark brown; rest of tarsus dark brown except basal 1/2 of 2nd tarsomere grayish-yellow; basitarsus (Fig. 1E) nearly parallel-sided from base to middle, then slightly narrowed toward apex, 6.16–6.75 times as long as its greatest width, and 0.77–0.83 and 0.63–0.66 times as wide as hind tibia and femur, respectively; calcipala well developed, nearly as long as wide; pedisulcus well developed. Claw (Fig. 1F) with large basal tooth 0.49 times as long as claw. Wing. Length 2.6–2.8 mm. Costa with 2 parallel rows of dark brown spinules and dark hairs except on subbasal portion near humeral cross vein with patch of whitish hairs. Subcosta with dark hairs except apical 1/4 bare. Basal portion of radius fully haired; R1 with dark spinules and hairs; R2 with dark hairs. Hair tuft on stem vein dark brown. Basal cell absent. Abdomen. Basal scale ocherous, with fringe of whitish-yellow long hairs. Dorsal surface of abdomen dark brown to brownish-black except basal 1/2 of segment 2 ocherous, moderately covered with dark brown hairs as well as whitish-yellow hairs; tergites 2 and 6–8 shiny when illuminated at certain angle of light; ventral surface of segment 7 with large sternal plate medially. Genitalia. Sternite 8 (Fig. 1G) wide, bare medially but furnished with 18–22 long hairs as well as few short hairs on each side. Ovipositor valves (Fig. 1G) triangular, thin, membranous except inner margin narrowly sclerotized, densely covered with microsetae interspersed with 5–9 short hairs; inner margins slightly concave medially and narrowly or moderately separated from each other. Genital fork (Fig. 1H,I) of inverted Y-form, with well sclerotized stem and relatively wide arms; each arm with lateral plate bearing round or triangular lobe-like projection directed medioposteriorly and short narrow stout projection directed anterodorsally; lateral plate of each arm with strongly sclerotized portion on anterior margin subapically in form of narrow horizontal bar, from which anterodorsally-directed projection arises. Paraproct in ventral view (Fig. 1J) roughly triangular, slightly longer than its greatest width; anteromedial surface nearly transparent, with 4–6 sensilla; paraproct in lateral view (Fig. 1K) somewhat protruding ventrally beyond ventral margin of cercus, and with 22–27 medium to long hairs on ventral and lateral surfaces. Cercus in lateral view (Fig. 1K) rounded posteriorly, 0.49 times as long as basal width. Spermatheca (Fig. 1L) nearly ovoidal, 1.15 times as long as its greatest width, strongly sclerotized except small area around juncture with duct and duct itself unsclerotized, with distinct reticulate surface pattern and without internal setae; main spermathecal duct narrow, somewhat narrower than both accessory ducts.

##### Male.

**Figure 2.**
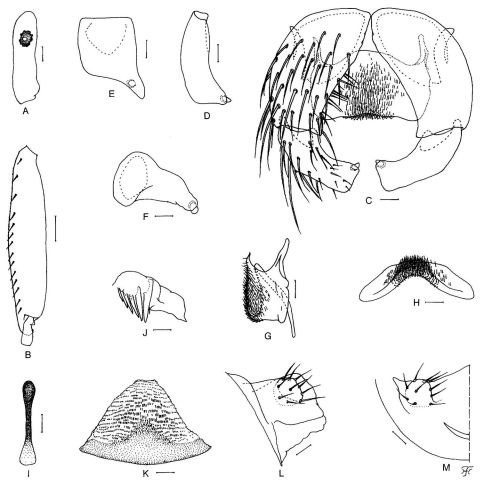
Male of Simulium (Nevermannia) *maeaiense* sp. n. **A** 3rd segment of maxillary palp with sensory vesicle (right side, front view) **B** basitarsus and 2nd tarsomere of left hind leg (outer view) **C** coxites, styles and ventral plate (ventral view) **D–F** right styles (**D** medial view; **E** ventrolateral view; **F** end view) **G** ventral plate and median sclerite (lateral view) **H** ventral plate (end view) **I** median sclerite (ventral view) **J** left paramere with 6 hooks (end view) **K** aedeagal membrane and dorsal plate (end view) **L, M** right half of 10th abdominal segments with cercus (**L** lateral view; **M** end view). Scale bars. 0.1 mm for B; 0.02 mm for A & C–M.

Body length 2.6–2.9 mm. Head. Slightly wider than thorax. Holoptic; upper eye consisting of large facets in 19 vertical columns and 20 horizontal rows. Clypeus brownish-black, not shiny, whitish pruinose, moderately covered with yellow hairs intermixed with dark brown longer hairs except medial portion widely bare. Antenna composed of scape, pedicel and 9 flagellomeres, dark brown except base of 1st flagellomere yellow, scape and pedicel light brown; 1st flagellomere elongate, 2.29–2.33 times as long as 2nd one. Maxillary palp dark brown, composed of 5 segments, proportional lengths of 3rd, 4th, and 5th segments 1.00:1.03–1.12:1.93–1.95; 3rd segment (Fig. 2A) of moderate size; sensory vesicle (Fig. 2A) small, ellipsoidal, 0.17–0.25 times as long as 3rd segment. Thorax. Scutum medium to dark brown, with 3 dark longitudinal vitae (1 medial and 2 submedial), whitish pruinose except 3 longitudinal vittae non-pruinose, and slightly shiny when viewed at certain angle of light, densely covered with golden-yellow recumbent hairs interspersed with several dark brown upright hairs on prescutellar area. Scutellum ocherous, with many dark brown upright long hairs as well as golden-yellow shorter hairs. Postnotum dark brown and bare. Pleural membrane bare. Katepisternum longer than deep, medium to dark brown, and bare. Legs. Foreleg: coxa yellow; trochanter yellow with somewhat dark portion on outer surface; femur dark yellow to light brown except apical cap dark brown; tibia dark brown though median large area on outer surface light to medium brown; tarsus entirely brownish-black; basitarsus slender, cylindrical, 10.0 times as long as its greatest width. Midleg: coxa medium brown though dark brown on posterolateral surface; trochanter yellow with somewhat dark portion on outer surface; femur dark yellow to light brown with apical cap dark brown; tibia dark brown with median large portion light brown; tarsus dark brown. Hind leg: coxa light to medium brown; trochanter yellow; femur dark yellow to light brown with apical cap dark brown; tibia dark brown with extreme base dark yellow and median large portion light to medium brown; tarsus medium brown except base and apical portion of basitarsus dark brown, and basal 1/2 of 2nd tarsomere light brown; basitarsus (Fig. 2B) enlarged, spindle-shaped, 4.63–4.86 times as long as its greatest width, and 0.88–0.92 and 0.85–0.86 times as wide as greatest widths of hind tibia and femur, respectively; calcipala well developed, nearly as long as basal width; pedisulcus moderately developed. Wing. As in female except subbasal patch of pale hairs on costal vein indistinct, and subcosta bare; length 2.4–2.5 mm. Abdomen. Basal scale brownish-black, with fringe of pale long hairs. Dorsal surface of abdominal segments entirely dark brown to brownish-black, not shiny, and moderately covered with light brown to black short to long hairs and yellow short hairs. Genitalia. Coxite in ventral view (Fig. 2C) rectangular, 1.92 times as long as its greatest width. Style in ventral view (Fig. 2C) short, 0.72 times as long as coxite, bent inwardly, with outer margin angled medially and with short stout spine apically; style in medial view (Fig. 2D) gently curved dorsally and nearly parallel-sided; style in ventrolateral view (Fig. 2E) broad, nearly parallel-sided from base to little beyond middle, then abruptly tapered apically; style in end view (Fig. 2F) tapered inward, with round apex. Ventral plate in ventral view (Fig. 2C) lamellate, subquadrate, 0.52 times as long as its greatest width, well sclerotized except anteromedian portion unsclerotized, with posterior margin slightly concave medially and submedially, and moderately covered with fine short setae on ventral surface except lateral portions widely bare; arm (Fig. 2C) short, slender, directed anteriorly; ventral plate in lateral view (Fig. 2G) with ventral margin nearly straight and arm short, tapered anterodorsally; ventral plate in caudal view (Fig. 2H) with dorsal margin markedly concave, with fine short setae centrally on posterior surface. Median sclerite (Fig. 2G,I) simple, club-shaped, narrow and strongly sclerotized except weakly sclerotized apical portion. Paramere (Fig. 2J) with 5 or 6 hooks decreasing in length toward apex. Aedeagal membrane (Fig. 2K) moderately covered with very minute setae; dorsal plate (Fig. 2K) triangular in shape, thin, weakly sclerotized. Ventral surface of 10th segment (Fig. 2L,M) without any distinct hairs near each posterolateral corner. Cercus (Fig. 2L,M) small, rounded and encircled by 8–12 simple hairs.

##### Pupa.

**Figure 3.**
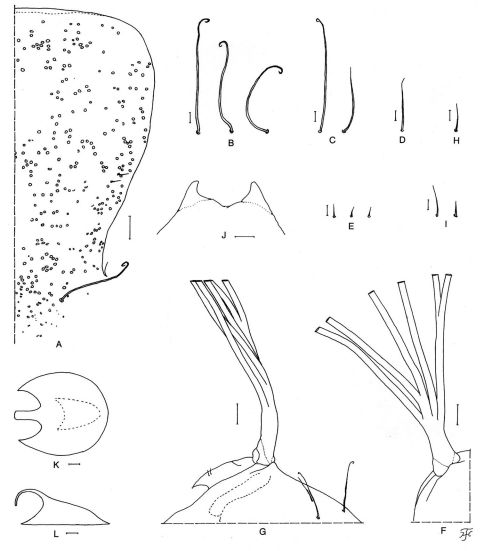
Pupa of Simulium (Nevermannia) *maeaiense* sp. n. **A** frons and part of face showing frontal and facial trichomes and tubercles (left half) **B–E** thoracic trichomes (**B** dorsomedial; **C** anterolateral; **D** mediolateral; **E** ventrolateral) **F, G** basal portions of left gill (**F** dorsal view; **G** lateral view) **H** short seta on dorsal surface of abdominal segment 1 **I** short seta and very short spinous seta on dorsal surface of abdominal segment 2 **J** terminal hooks (end view) **K, L** cocoons (**K** dorsal view; **L** lateral view). Scale bars. 0.5 mm for K, L; 0.1 mm for G, F; 0.05 mm for A; 0.02 mm for B–E & H–J.

Body length 3.0–3.5 mm. Head. Integument (Fig. 3A) dark yellow, moderately or sparsely covered with tubercles; antennal sheaths bare; frons with 2 very short simple trichomes (Fig. 3A) near lateral margin on each side; face with 1 long somewhat stout simple trichome with coiled apex (Fig. 3A) on each side. Thorax. Integument dark yellow, moderately (though sparsely on certain portions) covered with round tubercles, with 3 long somewhat stout simple trichomes with coiled apices (Fig. 3B) mediodorsally, 2 simple trichomes (1 long, somewhat stout, with coiled apex, and 1 medium-long, slender, with uncoiled apex) (Fig. 3C) anterolaterally, 1 medium-long somewhat stout simple trichome with uncoiled apex (Fig. 3D) mediolaterally, and 3 very short slender simple trichomes with uncoiled apices (Fig. 3E) ventrolaterally on each side. Gill (Fig. 3F,G) with 6 long thread-like slender filaments, arranged as 2+1+2+1 filaments in horizontal plane from inside to outside; common basal stalk of moderate length, subequal in length to interspiracular trunk, and stalk of inner pair medium-long to long (varying from 0.4 mm to 0.8 mm in individual pupae); stalk of middle pair very short to short; all filaments tapered toward tip, subequal in length (4.4–5.5 mm) and thickness to one another except 2 filaments of inner pair somewhat shorter (3.5–4.3 mm long) and slightly thinner than others; cuticular surface with distinct annular ridges and furrows though becoming less distinct near apex, and densely covered with minute tubercles. Abdomen. Dorsally, segments 1 and 2 ocherous, weakly tuberculate; segment 1 with 1 short slender simple seta (Fig. 3H) on each side; segment 2 with 1 short slender simple seta and 5 very short spinous setae (Fig. 3I) on each side; segments 3 and 4 each with 4 hooks and 1 very short spinous seta on each side; segments 5–8 each with spine-combs directed backward in transverse row and comb-like groups of minute spines (though comb-like groups of minute spines indistinct on segment 5) on each side; segment 9 with pair of distinct horn-shaped terminal hooks (Fig. 3J), comb-like groups of minute spines and few to several tubercles. Ventrally, segments 3–8 each with comb-like groups of minute spines; segment 4 with 4 very short simple slender setae, of which 1 somewhat spinous, on each side; segment 5 with pair of bifid hooks submedially and few very short slender setae on each side; segments 6 and 7 each with pair of bifid inner and simple outer hooks (though simple very short seta in place of simple outer hook on segment 7) and few slender very short setae on each side. Cocoon (Fig. 3K,L). Wall-pocket shaped, compactly woven without open spaces in web, thin, with anterior margin somewhat thickly woven, and extending ventrolaterally; anterodorsal projection long, 1.2–1.5 mm, extending forward but sometimes bent downward as shown in Fig. 3L; individual threads invisible; 3.8–4.2 mm long by 2.6–2.9 mm wide.

##### Mature larva.

**Figure 4.**
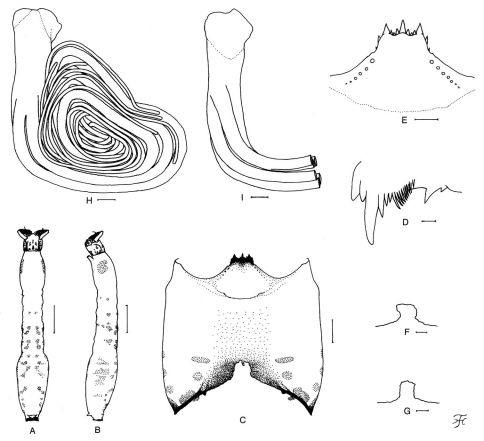
Mature larva of Simulium (Nevermannia) *maeaiense* sp. n. **A, B** whole body showing color markings on abdomen (**A** dorsal view; **B** lateral view) **C** head capsule showing small postgenal cleft (ventral view) **D** apical portion of mandible **E** hypostoma **F, G** postgenal cleft **H, I** histoblast of pupal gill (**H** left gill, outer view; **I** right gill, inner view; only basal portion shown). Scale bars. 1.0 mm for A & B; 0.1 mm for C; 0.05 mm for E–I; 0.01 mm for D.

Body length 7.0–7.8 mm. Body (Fig. 4A,B) yellow though somewhat grayish on ventral surface of thorax, with reddish-brown markings on abdomen; i.e., segment 1 on each side with 2 spots (1 laterally, 1 ventrolaterally), segment 2 on each side with 2 spots (1 dorsally, 1 laterally), segment 3 on each side with 6 spots (2 dorsally which are connected or separated, 3 laterally, 1 ventrolaterally), segment 4 on each side with 3 spots (2 dorsally which are connected or separated, 1 laterally), segment 5 on each side with 3 spots (2 dorsally, 1 laterally), segment 6 on each side with 3 spots (2 dorsally which are narrowly connected, 1 laterally), segment 7 on each side with 3 spots (2 dorsally which are usually connected, 1 laterally), segment 8 on each side with 2 spots dorsally, segments 5 and 6 each with faint spot ventromedially; lateral spots on segments 5–7 not well defined, some of other lateral spots very faint or even indistinct in some larvae. Cephalic apotome yellow, with distinct head spots. Lateral surface of head capsule yellow, except eye-spot region white; eyebrow distinct, with 1 dark round spot medially; 2 large spots and 1 small spot near posterior margin and 2 small spots below eye-spot region moderately positive. Ventral surface of head capsule (Fig. 4C) yellow except most of postgenal bridge light to dark brown and basal area on each side of postgenal cleft dark brown; 1 horizontal and 2 round spots on each side of postgenal cleft moderately positive. Cervical sclerite composed of 2 small elliptical pieces, not fused to occiput, widely separated medially from each other. Antenna consisting of 3 segments and apical sensillum, much longer than stem of labral fan; proportional lengths of 1st, 2nd, and 3rd segments 1.00:0.80–0.95:0.72–0.74. Labral fan with 23–25 main rays. Mandible (Fig. 4D) with mandibular serrations consisting of 2 teeth (1 large and 1 small); large tooth making nearly right angle or slightly less with mandible on apical side; comb-teeth composed of 3 teeth shortened from 1st to 3rd; supernumerary serrations absent. Hypostoma (Fig. 4E) with 9 apical teeth in row; median and corner teeth well developed; middle tooth of 3 intermediate teeth on each side smallest; lateral margin nearly smooth or with 1 or 2 very weakly developed teeth apically; 5–7 hypostomal bristles lying slightly divergent posteriorly from lateral margin on each side. Postgenal cleft (Fig. 4C,F,G) small, variable in shape, usually quadrate or rounded, 0.30–0.33 times as long as postgenal bridge. Thoracic cuticle bare; histoblast of pupal gill in outer view (Fig. 4H) with common basal stalk of medium length, from which 4 filaments arising; 2 middle filaments bearing very short stalk, and inner and outer filaments each arising individually; histoblast of pupal gill in inner view (Fig. 4I) showing remaining 2 filaments with relatively long stalk arising just below inner individual filament. Abdominal cuticle bare except both sides of anal sclerite moderately covered with simple colorless setae. Rectal scales present. Rectal organ compound, each lobe with 13–19 long finger-like secondary lobules. Anal sclerite of usual X-form, with anterior arms nearly as long as posterior ones, broadly sclerotized at basal juncture; sensilla absent on and just posterior to basal juncture area; accessory sclerite absent. Last abdominal segment much expanded ventrally forming large ventral papillae. Posterior circlet with 75–86 rows of up to 14 hooklets per row.

#### Type specimens.

Holotype female (with associated pupal exuviae and cocoon) (preserved in 80% ethanol) (QSBG 2010-45-24), reared from pupa, collected from a small slow-running stream (width 20 cm, depth 10 cm, water temperature 14.0°C, partially shaded, altitude 1,556 m, 20°04'43.2"N, 99°10'53.6"E), Doi Pha Hom Pok National Park, Mae Ai District, Chiang Mai Province, Thailand, 25.II.2010, by W. Srisuka and S. Suriva. Paratypes: 4 females, 5 males (with associated pupal exuviae and cocoon), all reared from pupae, and 10 mature larva, same data and date as those of holotype.

#### Biological notes.

The pupae and larvae of this new species were collected together with Simulium (Gomphostilbia) ceylonicum species-group sp. and Simulium (Simulium) doipuiense Takaoka and Choochote. The habit of biting of the females remains unknown.

#### Etymology.

The species name *maeaiense* refers to the name of the district, Mae Ai, where this new species was collected.

#### Remarks.

Simulium (Nevermannia) maeaiense sp. n. is readily assigned to the *feuerborni* species-group redefined by Takaoka (2003) by the combination of the following characteristics: male genitalia with a simple lamellate ventral plate (Fig. 2C), a short inwardly-twisted style (Fig. 2E), a simple narrow median sclerite (Fig. 2I) and several parameral hooks (Fig. 2J); the pupal gill with six long thread-like filaments per side (Fig. 3F); and the larval head with a small short postgenal cleft (Fig. 4C).

The female of Simulium (Nevermannia) maeaiense sp. n. is distinctive among the *feuerborni* species-group in that the cibarium has numerous minute cone-shaped processes on the lower part (Fig. 1C) and the genital fork bears a strongly sclerotized narrow horizontal bar at the base of the anterodorsally directed projection (Fig. 1H,I) on each arm. This combination of characters has not been reported in any known species of the species-group except two species, Simulium (Nevermannia) fruticosum from Thailand, and Simulium (Nevermannia) sasai (Rubtsov) from Japan, which have, respectively, 11 and 16 minute processes on the lower part of the cibarium, according to the illustrations given by Takaoka and Choochote (2005) and Sato et al. (2005).

The pupa of this new species is also remarkable within this species-group in having the unique arrangement of the gill filaments (Fig. 3F) (i.e., arranged as 2+1+2+1 filaments from inside to outside; two stalks of the inner and middle pairs and two individual filaments arising close together from the common basal stalk nearly at the same level and lying nearly in a horizontal plane).

On the other hand, the male of this new species is very similar to those of other known species in many features including the genitalia and barely distinguished by the combination of the numbers of the vertical columns (19) and horizontal rows (20) of the enlarged upper-eye facets and the number of parameral hooks (5 or 6).

The mature larva of this new species has reddish-brown markings on the abdomen as in most known species of the *feuerborni* species-group, but is easily distinguished from other species by possessing six colored spots on each side of the abdominal segment 3 (Fig. 4B).

In having the cocoon with an anterodorsal projection (Fig. 3K), Simulium (Nevermannia) maeaiense sp. n. appears to be somewhat similar to the following six known species: Simulium (Nevermannia) fangense from Thailand (Takaoka and Choochote 2006), Simulium (Nevermannia) feuerborni from Java, Sumatra, Peninsular Malaysia and Thailand (Edwards 1934; Kuvangkadilok et al. 1999; Takaoka and Davies 1995, 1996; Takaoka et al. 2000), Simulium (Nevermannia) leigongshanense Chen and & Zhang from China (Chen and Zhang 1997), Simulium (Nevermannia) mongarense Takaoka and Somboon from Bhutan (Takaoka and Somboon 2008), Simulium (Nevermannia) praelargum Datta from India (Datta 1973) and Simulium (Nevermannia) sasai from Japan (Sato et al. 2005). However, this new species is easily distinguished from these known species in the female by the cibarium and genital fork, in the pupa by the arrangement of the gill filaments, and in the larva by the reddish-brown markings on the abdomen, as already mentioned above.

This new species is distinguished from all six known species of the *feuerborni* species-group in Thailand by the following keys.

## Keys to seven species of the feuerborni species-group of the subgenus Simulium (Nevermannia) in Thailand

Females*

**Table:** 

1	Fore basitarsus 7.3–7.4 times as long as its greatest width; claw with basal tooth 0.42–0.43 times as long as claw	2
–	Fore basitarsus 7.7–8.4 times as long as its greatest width; claw with basal tooth 0.49–0.50 times as long as claw	3
2	Frons-head ratio 1.00:1.48	Simulium chiangklangense
–	Frons-head ratio 1.00:1.54	Simulium feuerborni
3	Maxillary lacinia with 10 or 11 inner teeth; mandible with 17 or 18 inner teeth	Simulium vessabutrae
–	Maxillary lacinia with 6–9 inner teeth; mandible with 20–23 inner teeth	4
4	Sensory vesicle 0.51–0.53 times as long as 3rd segment of maxillary palp; genital fork with a strongly sclerotized horizontal bar on each arm	Simulium sp. n.
–	Sensory vesicle 0.58–0.66 times as long as 3rd segment of maxillary palp; Genital fork without horizontal bar on each arm	5
5	Labrum 0.92 times as long as clypeus	Simulium fangense
–	Labrum as long as clypeus	Simulium fruticosum

* Simulium wichaii is not included because its female remains unknown.

Males**

**Table:** 

1	Scutum light to dark brown or reddish-brown, with 3 dark longitudinal vittae	2
–	Scutum brownish-black, without dark longitudinal vittae	4
2	Paramere with 3 or 4 hooks	Simulium feuerborni
–	Paramere with 5–7 hooks	3
3	Upper eye with large facets in 14 or 15 vertical columns and in 17 or 18 horizontal rows	Simulium fruticosum
–	Upper eye with large facets in 19 vertical columns and in 20 horizontal rows	Simulium maeaiense sp. n.
4	Upper eye with large facets in 21 vertical columns and in 21 horizontal rows	Simulium fangense
–	Upper eye with large facets in 14–17 vertical columns and in 16–18 horizontal rows	5
5	Upper eye with large facets in 14 vertical columns and in 16 or 17 horizontal rows; paramere with 7or 8 hooks	Simulium wichaii
–	Upper eye with large facets in 17 vertical columns and in 18 horizontal rows; paramere with 9 hooks	Simulium vessabutrae

** Simulium chiangklangense is not included because its male remains unknown.

**Pupae**

**Table:** 

1	Cocoon with anterodorsal projection	2
–	Cocoon without anterodorsal projection	4
2	Six gill filaments arranged as 2+1+2+1 lying nearly horizontally from inside to outward	Simulium maeaiense sp. n.
–	Six gill filaments arranged otherwise	3
3	Six gill filaments arranged as 4+2 from dorsal to ventral; stalk of ventral pair medium-long to long	Simulium feuerborni
–	Six gill filaments arranged otherwise; stalk of ventral pair short	Simulium fangense
4	Head and thorax covered with dark brown large tubercles	Simulium wichaii
–	Head and thorax covered with yellowish brown small tubercles	5
5	Six gill filaments arranged as 2+(2+2), each pair with medium-long to long stalk; common basal stalk long	Simulium vessabutrae
–	Six gill filaments arranged otherwise; common basal stalk short to medium-long	6
6	Six gill filaments arranged as 4+2 from dorsal to ventral; stalk of ventral pair medium-long	Simulium fruticosum
–	Six gill filaments arranged as 1+1+2+2 from inside to outside; stalks of pairs short	Simulium chiangklangense

Mature larvae***

**Table:** 

1	Ventral surface of head capsule not darkened on postgenal bridge	2
–	Ventral surface of head capsule darkened on postgenal bridge	3
2	Labral fan with 16 main rays	Simulium wichaii
–	Labral fan with 28 main rays	Simulium vessabutrae
3	Postgenal cleft 0.30–0.43 times as long as postgenal bridge	4
–	Postgenal cleft 0.50–0.64 times as long as postgenal bridge	5
4	Posterior circlet with 75–86 rows of up to 14 hooklets per row	Simulium maeaiense sp. n.
–	Posterior circlet with about 90 rows of up to 16 hooklets per row	Simulium feuerborni
5	Each lobe of rectal organ with 11 or 12 secondary lobules	Simulium fruticosum
–	Each lobe of rectal organ with 17–21 secondary lobules	Simulium fangense

***Simulium chiangklangense is not included because its larva remains unknown.

## Supplementary Material

XML Treatment for Simulium (Nevermannia) maeaiense
